# Kinetically-derived maximal dose (KMD) indicates lack of human carcinogenicity of ethylbenzene

**DOI:** 10.1007/s00204-023-03629-7

**Published:** 2023-12-07

**Authors:** Lyle D. Burgoon, Christopher J. Borgert, Claudio Fuentes, James E. Klaunig

**Affiliations:** 1 Raptor Pharm & Tox, Ltd, Apex, NC USA; 2 Applied Pharmacology and Toxicology, Inc., University of Florida College of Veterinary Medicine, Gainesville, FL USA; 3 Oregon State University, Corvallis, OR USA; 4 Indiana University School of Public Health, Bloomington, IN USA

**Keywords:** Kinetic Maximum Dose, Ethylbenzene, Carcinogen, Threshold, Regulatory Policy, Pharmacokinetics/Toxicokinetics

## Abstract

The kinetically-derived maximal dose (KMD) is defined as the maximal external dose at which kinetics are unchanged relative to lower doses, e.g., doses at which kinetic processes are not saturated. Toxicity produced at doses above the KMD can be qualitatively different from toxicity produced at lower doses. Here, we test the hypothesis that neoplastic lesions reported in the National Toxicology Program’s (NTP) rodent cancer bioassay with ethylbenzene are a high-dose phenomenon secondary to saturation of elimination kinetics. To test this, we applied Bayesian modeling on kinetic data for ethylbenzene from rats and humans to estimate the *V*_max_ and *K*_m_ for the Michaelis–Menten equation that governs the elimination kinetics. Analysis of the Michaelis–Menten elimination curve generated from those *V*_max_ and *K*_m_ values indicated KMD ranges for venous ethylbenzene of 8–17 mg/L in rats and 10–18 mg/L in humans. Those venous concentrations are produced by inhalation concentrations of around 200 ppm ethylbenzene, which is well above typical human exposures. These KMD estimates support the hypothesis that neoplastic lesions seen in the NTP rodent bioassay occur secondary to saturation of ethylbenzene elimination pathways and are not relevant for human risk assessment. Thus, ethylbenzene does not pose a credible cancer risk to humans under foreseeable exposure conditions. Cancer risk assessments focused on protecting human health should avoid endpoint data from rodents exposed to ethylbenzene above the KMD range and future toxicological testing should focus on doses below the KMD range.

**Supplementary Information:**

The online version contains supplementary material available at 10.1007/s00204-023-03629-7.

## Introduction

Risk assessors are tasked with characterizing and quantifying risks and identifying chemical exposure levels that are safe for humans but in doing so, are required by regulatory agencies to consider data from animals exposed up to the maximum tolerated dose (MTD). Typically, the MTD not only exceeds anticipated human exposure levels but frequently exceeds dose levels that saturate drug and chemical metabolism and elimination pathways in the test species, leading to a host of toxic effects that are irrelevant to the effects expected from lower concentrations where kinetic processes^1^ are not saturated (Borgert et al. 2021; Burgoon et al. 2022; Bus 2017; Andersen 1981). In addition, effects seen at high dose levels provide false information on the mechanism of action by which the compound is producing the toxic effect. Thus, we have argued that MTD testing is biologically irrelevant to human exposures and therefore an unethical waste of animals and resources (Borgert et al. 2021). Instead, we contend that toxicity testing should be constrained to the kinetically-derived maximum dose (KMD).

We define the KMD as the maximal external dose where kinetics are unchanged relative to lower doses (Borgert et al. 2021). In practice, we estimate the KMD as a range to represent our uncertainty about the precise location of the KMD (Burgoon et al. 2022). The KMD has practical importance for the identification of safe exposure levels to protect against tumorigenesis and cancers. Consider a chemical that increases tumorigenicity in rodent test species at “high” doses but shows no increase in tumor incidence at lower doses. Although potentially an issue of test sensitivity, this might instead be an example where saturation of elimination kinetics is a requisite threshold step preceding the carcinogenic mechanism. As the elimination rate reaches its maximum at doses significantly above the KMD, the chemical concentration in blood is likely to increase and may exceed a threshold concentration that favors selective lesion growth and neoplastic transformation. That chemicals induce tumor formation through a threshold mechanism is well-supported by numerous lines of scientific evidence (Golden et al. 2019).

Ethylbenzene is a volatile organic chemical commonly used as feedstock for the synthesis of styrene. It may also be found in paints, varnishes, and many other products, including as a component of mixed xylenes (Saghir et al. 2010). The National Toxicology Program conducted a 2-year cancer bioassay with ethylbenzene in rats and mice that showed no excess tumors at concentrations of 75 and 250 ppm ethylbenzene in air, but potentially significant increases at 750 ppm. In male rats exposed to 750 ppm ethylbenzene vapors for 6 h per day, 5 days per week for 104 weeks, the incidence of renal tubule adenoma and adenoma/carcinoma combined was reported to be increased relative to concurrent controls, as was the incidence of renal tubule hyperplasia. Renal tubule adenoma and hyperplasia were reported to be increased in both males and females exposed to 750 ppm and the severity of nephropathy was reportedly increased in males exposed to 750 ppm ethylbenzene and in all exposed females (75, 250 and 750 ppm). In rats exposed to 750 ppm, an increase was reported in interstitial cell testis adenoma relative to concurrent controls which slightly exceeded the historical control range for NTP inhalation studies (NTP 1999).

In mice exposed to 750 ppm ethylbenzene vapors for 6 h per day, 5 days per week for 104 weeks, the incidence of alveolar/bronchiolar adenoma and alveolar/bronchiolar adenoma or carcinoma (combined) were reported to be significantly greater than those in the concurrent chamber control group but within the historical control range for NTP studies. Alveolar epithelial metaplasia was reportedly increased in males of that group relative to concurrent controls, but not in males exposed to 75 or 250 ppm ethylbenzene. In females exposed to 750 ppm ethylbenzene, the incidence of hepatocellular adenoma and adenoma/carcinoma (combined) were reported to be increased relative to concurrent chamber controls, but within the range for historical controls (NTP 1999). There were a variety of nonneoplastic liver changes reported in male mice exposed to ethylbenzene, including syncytial alteration of hepatocytes, hepatocellular hypertrophy and hepatocyte necrosis. Hyperplasia of the pituitary gland pars distalis and incidence of thyroid gland follicular cell hyperplasia were reported to be significantly increased relative to concurrent controls in 750 ppm males and in females exposed to 250 and 750 ppm ethylbenzene. Genotoxicity tests generally failed to identify a clear genotoxic potential for ethylbenzene (IARC 2000; NTP 1999).

Based on these observations and previous kinetic modeling (Saghir et al. 2010; Charest-Tardif et al. 2006) indicating that ethylbenzene metabolism becomes saturated at inhalation exposure concentrations of 500 ppm, we hypothesized that the increase in tumor incidence observed at 750 ppm ethylbenzene is consistent with toxicity that occurs secondary to saturation of elimination kinetics rather than to frank carcinogenicity and that a KMD likely exists between 200 and 500 ppm. A KMD estimate below 750 ppm would suggest that the tumors and cancers observed in the NTP bioassay are likely not relevant to lower exposure levels, whereas a KMD estimate in the range of 750 ppm or greater would refute our hypotheses and would suggest that saturation of elimination kinetics does not contribute to tumorigenicity and carcinogenicity. Here, we estimate KMD ranges for ethylbenzene based on kinetic data from rats and humans and interpret those data in the context of the rodent tumors observed and for human cancer risk assessment.

## Methods

### Literature search

We conducted a comprehensive literature search to identify studies that contain kinetic data for ethylbenzene in mice, rats, and humans. To accomplish this, the literature searches were conducted in medical scientific databases (Pubmed, EMBASE, Web of Science) and regulatory websites/databases (NIOSH, USEPA, ATSDR, National Toxicology Program, NTIS). The search strategies linked Ethylbenzene (RN#100-41-4) with focused key terms: Pharmacokinetics; Kinetic Maximum Dose (KMD); Toxicokinetics; Absorption, Distribution, Metabolism and Excretion (ADME; Physiologically based pharmacokinetic modeling (PBPK); Physiologically based toxicokinetic (PBTK) models. In addition to those linked terms, broader search terms associated with “toxicity, health risk assessment, inhalation toxicity” were used identify general toxicity studies of ethylbenzene that might include relevant PK/TK data.

From this comprehensive literature search, we identified 58 potentially useful peer-reviewed articles and reports (see Supplemental Materials) and upon review of those publications, narrowed this list to 5 peer-reviewed articles that contained sufficient kinetic data to support a valid KMD model. Those data were from rats and humans. We found the available mouse data (Charest-Tardiff et al. 2006; Fuciarelli et al. 2000) to be insufficient for a reliable estimation of the Michaelis constants *V*_max_ and *K*_m_ for mice, but also determined that the rat kinetic data are applicable to mice, which is consistent with conclusions of Nong et al. (2007).

### KMD range estimation

We used a Bayesian approach to estimate the *K*_m_ and *V*_max_ for the system-wide Michaelis–Menten function that governs the kinetic curve (Burgoon et al. 2022). Briefly, this involved applying Bayesian analysis with differential equations to information from published, peer-reviewed studies on ethylbenzene kinetics to build statistical distributions of plausible values of the *K*_m_ and *V*_max_ for ethylbenzene elimination. From those distributions of likely *K*_m_ and *V*_max_ values, a set of Michaelis–Menten equations were generated that are likely to represent the slope function for the relationship between ethylbenzene exposure and blood concentration. The resulting Michaelis–Menten functions were then investigated using a change-point methodology known as the “kneedle” algorithm (Burgoon et al. 2022; Satopaa et al. 2011) to identify the possible KMD range, which we have defined as the range of administered concentrations at which maximum curvature has been achieved. The KMD range is thus the region of the curve that begins to approach an asymptote.

We validated our *K*_m_ and *V*_max_ using “out of sample data”. This involves comparing the kinetic measurements for a concentration of ethylbenzene not used to estimate the *K*_m_ and *V*_max_ for the system-wide Michaelis–Menten function (we call those data and resulting curve “ground truth”) with the curve generated from the estimated *K*_m_ and *V*_max_ for the system-wide Michaelis–Menten function. Specifically, we used kinetic data for 50 ppm ethylbenzene to estimate a system-wide *K*_m_ and *V*_max_ and then compared the kinetic curve generated from those parameters with the kinetic curve for 100 ppm ethylbenzene at 4, 4.5, 5, 5.5, and 6 h post exposure (ground truth). If the kinetic curve generated from the estimated *K*_m_ and *V*_max_ fit the ground truth measurements sufficiently well, we would conclude that the model produced a valid result. We would conclude the model to be invalid if the kinetic curve generated from the estimated *K*_m_ and *V*_max_ did not fit the ground truth measurements. We defined “sufficient fit” as a root mean square error (RMSE) less than 0.05 units based on observed values compared with mean predicted values from the model. In this case, an RMSE of 0.0126 units was observed, indicating that the model produced sufficient fit to conclude a valid result.

## Results

Table 1 lists the five peer-reviewed publications containing data used here to determine the KMD for rats and humans.

**Table 1:** Peer-reviewed publications used to determine the KMD for rats and humans

Publication	Species
Haddad et al. (1999) Toxicology and Applied Pharmacology 161: 249–257	Rat
Haddad et al. (2000) Toxicology and Applied Pharmacology 167: 199–209	Rat
Freundt et al. (1989) Bull. Environ. Contam. Toxicol. 42: 495–8	Rat
Tardif et al. (1999) Toxicology and Applied Pharmacology 144: 120–134	Rats and Human
Marchand et al. (2015) Toxicological Sciences 144(2): 414–24	Human

As described in “Materials and methods”, the method of Burgoon et al. (2022) was used to estimate likely distributions of *K*_m_ and* V*_max_ representing likely Michaelis–Menten functions for ethylbenzene, and the kneedle algorithm was then used to investigate the resulting Michaelis–Menten curves to identify the likely KMD range. We used the male rat venous ethylbenzene concentration data at an external exposure of 50 ppm (Haddad et al. 1999, 2000) to estimate the *K*_m_ and *V*_max_ of the global kinetic system (Burgoon et al. 2022). Blood measurements were taken at 4, 4.5, 5, 5.5, and 6 h post exposure.

**Fig. 1:**
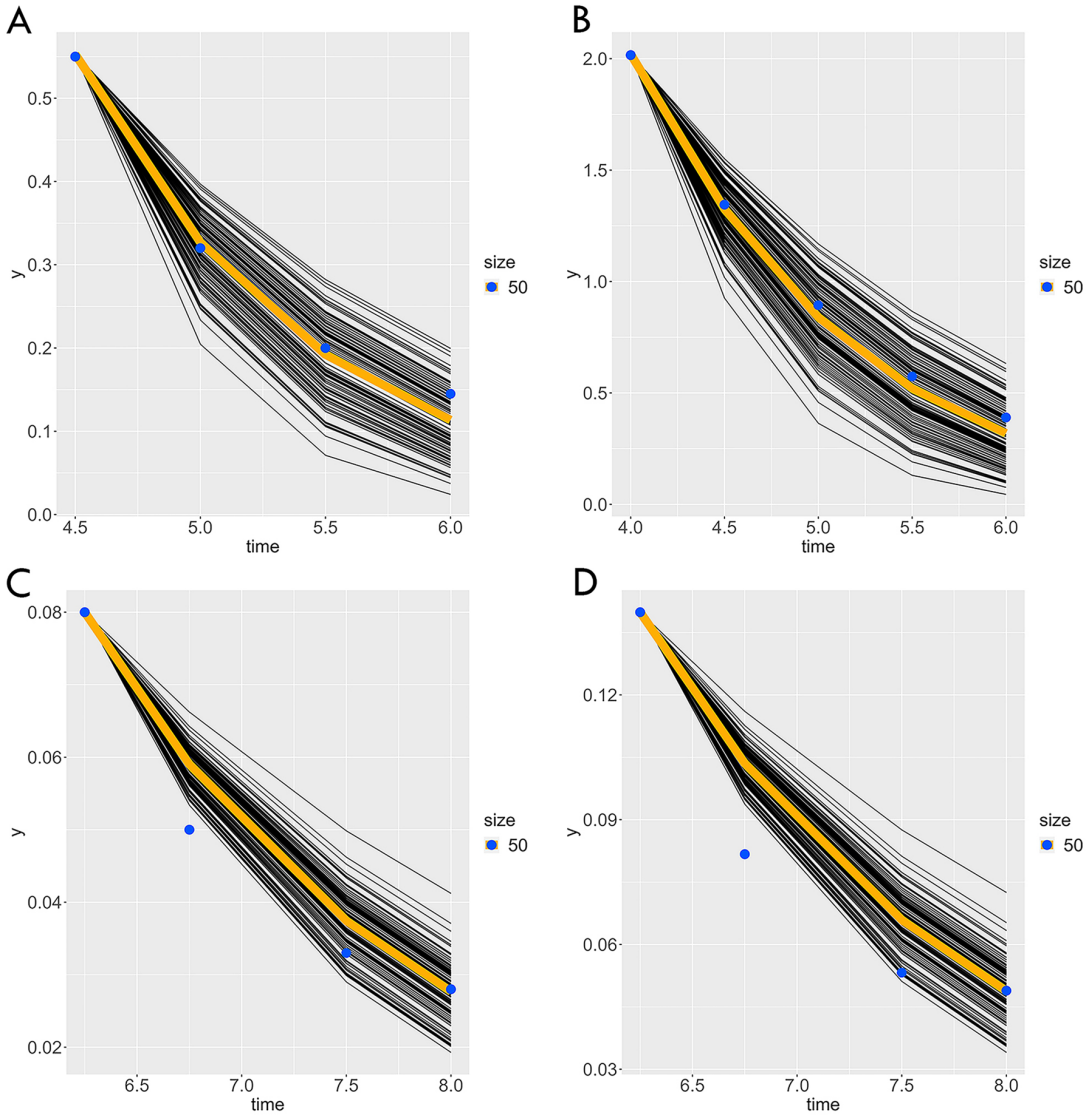
**A** Rat 50 ppm exposure, **B** rat 100 ppm exposure (validation), **C** human 12.5 ppm exposure, **D** human 25 ppm exposure (validation). Spaghetti plots show 100 different runs; the yellow line is the mean of the 100 runs, and the blue dots are the measured venous ethylbenzene concentrations

The estimated *K*_m_ ranged from 3.49–5.01 mg/L, while the estimated *V*_max_ ranged from 3.39 to 7.64 mg/(L*30 min) or 0.11 to 0.25 mg/L min (note: our model runs in 30 min increments, so 3.39–7.64 mg/(L*30 min) is the output from our model; however, we are reporting mg/L*min for reader convenience). The estimated *K*_m_ and *V*_max_ for rat at the 50 ppm exposure in rats resulted in a kinetic curve that matched the kinetic measured data (i.e., the measured data as blue dots overlay the yellow line, which is the mean of the 100 model runs; Fig. 1A), indicating a valid model result.

We validated the *K*_m_ and *V*_max_ estimates from the 50 ppm ethylbenzene exposure group by comparing those to the out of sample ground truth data for rats exposed to 100 ppm ethylbenzene (Fig. 1B). Again, we see that the measured data (the blue dots in Fig. 1B) overlay the mean of the 100 model runs (the yellow line in Fig. 1B). This provides increased confidence that the rat *K*_m_ and *V*_max_ estimates are valid estimates independent of the exposure concentrations.

Using the validated and estimated *K*_m_ and *V*_max_ values, we estimated the rat KMD range to be from 8 to 17 mg/L venous ethylbenzene concentration. Interpolation of experimental data published by Haddad et al. (1999; see Table 3 therein) indicates that this venous blood concentration is produced in rats following 4 h inhalation exposure to 200 ppm ethylbenzene. We used the *K*_m_ and *V*_max_ estimates to generate the Michaelis–Menten curve that allowed us to calculate the KMD range using the kneedle algorithm.

The human *K*_m_ and *V*_max_ was estimated by the same approach used for estimating the rat *K*_m_ and *V*_max_ (Burgoon et al. 2022), but human venous ethylbenzene concentration data were used from 6.25, 6.75, 7.50, and 8.0 h post 12.5 ppm inhalation exposure provided by Marchand et al. (2015). The human data also approximated the estimated curve with RMSE > 0.05 units, indicating a valid model result (Fig. 1C). The *y*-axis is particularly important in considering the human kinetic curves in Fig. 1C/D. Typically, the model overpredicts the true value by 0.01 mg/L or less, which is well within our stated tolerance for a valid model result. Since fewer data were used to estimate the human *K*_m_ and *V*_max_ compared to the rat, it is possible that the model would overpredict less if more human data were available. Regardless, the overprediction is so small that the impact on the KMD range would be negligible.

*K*_m_ and *V*_max_ estimates were used to generate the Michaelis–Menten curve, and the kneedle algorithm was applied to estimate a human KMD range (Burgoon et al. 2022) of 10–18 mg/L venous ethylbenzene. Owing to limitations in the existing literature, this venous ethylbenzene concentration could not be correlated to an external exposure concentration. However, assuming that the kinetics of ethylbenzene absorption are similar in humans and rats, as suggested by Marchand et al (2015), one would expect an external exposure of 200 ppm over 4 h to exceed the human KMD.

## Discussion

Kinetic data for ethylbenzene from rats and humans were used to estimate KMD ranges for each species. The KMD range in rats was estimated to be from 8 to 17 mg/L venous ethylbenzene, and for humans, from 10 to 18 mg/L venous ethylbenzene. These ranges correspond to an inhalation concentration of approximately 200 ppm ethylbenzene.

Our results support the conclusion of Saghir et al. (2010), that saturation of ethylbenzene elimination occurs between 200 and 500 ppm in rodents, and with Charest-Tardif et al. (2006), that ethylbenzene exposures of between 200 and 500 ppm saturates metabolism and elimination kinetics in rats. Our results are also consistent with the data from the NTP ethylbenzene bioassay results, which showed that neoplastic lesions in the rodent cancer bioassay occurred at 750 ppm, but not at lower concentrations. However, our results contradict the NTP conclusion that the reported ethylbenzene-associated rodent tumors are human relevant. Given that the KMD range is around 200 ppm in both rats and humans, our results support the conclusion that cancer is seen only at concentrations that exceed enzymatic saturation and where saturation of clearance mechanisms is apparent in both rats and humans.

In mice, tumors have also been reported following chronic exposure to ethylbenzene. Unfortunately, the available mouse data (Charest-Tardiff et al. 2006; Fuciarelli et al. 2000) needed for proper KMD estimation was determined to be insufficient for a reliable estimation of the Michaelis constants *V*_max_ and *K*_m_ for mice. This is consistent with determinations made previously by Nong et al. (2007). The kinetic profile for ethylbenzene in mice clearly differs between 75 ppm (linear) and 750 ppm (saturated), prompting the authors who reported this phenomenon to conclude that the kinetics become saturated at an intermediate concentration not greater than 500 ppm (Charest-Tardiff et al. 2006). Based on Nong et al.’s (2007) validation of a PBPK model for ethylbenzene in mice that had used Michaelis constants derived from rat, our KMD estimate should be equally applicable for interpreting the results of toxicology and carcinogenicity studies conducted in mice and rats.

The reliance of our method on the Michaelis constants *K*_m_ and *V*_max_ is a unique strength that, combined with corroborative mechanistic information, avoids the challenges often associated with application of kinetics in dose-setting and interpretation of toxicological findings, such as those discussed by Tan et al., (2021). These constants are unassailable fundamentals of biotransformation and elimination kinetics that allow KMDs to be established without reliance on Area Under the blood Concentration curve (AUC), which can be less precise than Michaelis constants (Burgoon et al. 2022). AUC data on ethylbenzene are unavailable for derivation of the KMD for ethylbenzene in rodents generally. The use of rat data was necessary for our purposes because kinetic data from mouse are as yet insufficient to derive Michaelis constants. Nonetheless, generalizing from rat to mouse is justified for this purpose as demonstrated by Nong et al. (2007), who used rat kinetic data on ethylbenzene to develop a PB/PK that was validated to be applicable to mouse.

It is important to appreciate the conservative nature of a KMD range that is estimated by the kneedle algorithm as we apply it (Burgoon et al. 2022). Because our method estimates the KMD range based on the region of the ethylbenzene exposure/blood concentration curve that approaches an asymptote, it does not utilize information from the area of the curve at which the rate of change in slope begins to increase. In other words, because our method relies on *V*_max_, it identifies the end of the curve, not the beginning or the mid-point of the curve. The beginning of the curve, however, may be biologically important because it indicates the range in which the relationship between exposure and blood concentration begins to change in a biologically meaningful way. Thus, it could be argued that either the beginning of the curve or the mid-point of the curve more accurately reflects a biologically meaningful KMD range than the end of the curve, and thus, that our method is overly conservative.

With this conservatism in mind, it is clear that our KMD estimate supports the argument that all cancers, tumors, and potential pre-neoplastic lesions identified in the NTP carcinogenicity study of ethylbenzene in rodent occur secondary to kinetic changes that occur in the range of 200 ppm inhalation exposure. Mechanistic factors underlying the tumorigenic activity of ethylbenzene have been investigated following inhalation exposure of F344 rats and B6C3F1 mice at 75 and 750 ppm for 6 h per day, 5 days per week, for 1 or 4 weeks (Stott et al. 2003). Exposure to the nontumorigenic concentration—75 ppm—produced few changes in organ weights, mixed function oxygenase activity, glucuronosyl transferase activities, S-phase DNA synthesis, apoptosis, α2u-globulin deposition, or histopathology. The effects differed between males and females but were generally confined to the 750 ppm exposure level. The results indicate that exposure to high, but not low, levels of ethylbenzene by inhalation can cause changes in rat kidneys characterized by acceleration of chronic progressive nephropathy (CPN) in males and females and α2u-globulin deposition in males, and mouse liver and lungs consistent with a nongenotoxic mode of tumorigenic action that is dependent on cell proliferation and alterations in the dynamics of various cell populations in target tissues (Ashby et al. 1994; Stott et al. 2003). Exacerbation of rat-specific CPN has been shown to lack a human counterpart (Hard et al. 2009) and thus, increases in the incidence of CPN-related and α2u-globulin-related renal tumors induced by ethylbenzene should not be used for human risk assessment as neither mode of action has qualitative relevance to humans.

Thus, the modes of action that likely lead to tumorigenic effects in rodents are operative at exposure levels that exceed the KMD range, but not at exposures below the KMD range. Consistent with numerous other examples of dose-dependent changes in mechanisms of toxicity (e.g., Slikker et al. 2004), a phenomenon so common it would appear to be the rule rather than the exception, this strongly suggests that effects observed following exposure to ethylbenzene concentrations above its KMD range would not be relevant for assessing cancer hazards in rodents or in humans exposed to concentrations below the KMD, since rodents and humans have similar KMD ranges. Such effects would include in rats: renal tubule adenoma and adenoma/carcinoma combined and renal tubule hyperplasia in male rats, renal tubule adenoma and hyperplasia in males and females, nephropathy in males and females, and interstitial cell adenoma in male testis (NTP 1999). In mice, these include: alveolar/bronchiolar adenoma and alveolar/bronchiolar adenoma or carcinoma (combined), alveolar epithelial metaplasia in males, hepatocellular adenoma and adenoma/carcinoma (combined) in females, syncytial alteration of hepatocytes, hepatocellular hypertrophy and hepatocyte necrosis in males, hyperplasia of the pituitary gland pars distalis and incidence of thyroid gland follicular cell hyperplasia in males and in females.

It has been argued that evidence of alveolar carcinoma was observed in mice at concentrations lower than those required to produce liver and kidney tumors. However, the incidence of frank alveolar carcinoma was not observed at any level of exposure in mice, and combined adenoma/carcinoma incidence was statistically elevated only at 750 ppm, but not at 250 ppm or 50 ppm exposure in studies conducted by the U.S. National Toxicology Program (NTP 1999). Furthermore, the alveolar precursor lesions alleged by NTP to be observable at lower concentrations are dependent on mouse-lung-specific metabolism of ethylbenzene (discussed in Nong et al. 2007). As with styrene (Cruzan et al. 2002), the higher conversion of ethylbenzene to CYP2E1 metabolites in mouse lung are likely responsible for changes observed in mouse lung at exposures below 200 ppm, but these do not appear to correspond with neoplasia or tumors at higher concentrations. Such pulmonary effects in mice are highly unlikely to be relevant to humans since the pulmonary activity of CYP2E1 in mice is approximately 20-fold higher than in mouse liver, and 23 and 600 times higher in mouse versus rats and human lung microsomes. In addition, the human relevance of the mouse lung adenomas is questionable (Cohen et al. 2020).

Despite this evidence, Huff et al., (2010), assert that cancers associated with ethylbenzene exposure in the rodent cancer bioassay are human relevant, even though it is clear from the data in the NTP report on inhalation exposure to ethylbenzene that neoplastic lesions were only seen in the group exposed to the highest concentration of 750 ppm, but not at 250 ppm or below (National Toxicology Program 1999). In contrast, Saghir et al. (2010) argued that these neoplastic lesions are consistent with high exposure levels of ethylbenzene leading to metabolic saturation. Scientists from the NTP countered that there are no high dose phenomena because they detected *“[s]ignificant dose response trends”*, and that the saturation argument is insufficient as they would “*not expect to see much increase in tumor incidence in the top exposure [750 ppm] compared to the mid-level exposure [250 ppm], because both exposures are in the ‘saturation zone.”*

Huff et al.’s (2010) reliance on “trend significance” has been termed abusive statistics (Wood et al. 2014) and must be avoided. Among the many problems with this method, it treats estimates of population effects at each concentration point as being both precise and accurate, which does not comport with good statistical practice, especially regarding fundamentals of sampling theory. Obtaining a precise, accurate reproduction of the population incidence rate for neoplastic lesions would be highly unlikely based on group sizes of 50 animals, especially when that rate is likely very small. A simple simulation using a beta-binomial distribution clearly demonstrates this point.

NTP argues that if carcinogenic transformation were dependent on saturation of elimination kinetics, tumor incidence would be similar at 250 ppm and 750 ppm rather than markedly increased at 750 ppm relative to 250 ppm, since both concentrations are within the “saturation zone” (Huff et al. 2010). That argument, however, betrays a misunderstanding of Michaelis–Menten kinetics with respect not only to saturation but also to its relationship to the mode of carcinogenic action. The issue is akin to the classic calculus problem of over-filling a bathtub, where overtopping the walls of the bathtub will cause real and significant problems, analogous to induction of a carcinogenic mode of action. A bathtub will drain at a constant rate whenever water flows into the tub at a rate equal to or greater than the drain capacity; this is analogous to saturation of elimination pathways in an animal. If the drain capacity is 200 mL/min and you are filling the bathtub at 250 mL/min, then every minute, the tub accumulates 50 mL. Let’s also assume your bathtub walls are such that it can handle 5 L/day. If you fill the bathtub for 15 min each day, then at 250 mL/min, you will never overtop the bathtub. But, at 750 mL/min you will overfill the bathtub each and every day by 3.25 L. Biologically speaking, the walls of the bathtub are the threshold required for the carcinogenic mode of action—in other words, you need to overtop the bathtub in order to activate that mode of action. So, even though the bathtub is saturated at 200 mL/min and cannot drain faster than that, it is the walls of the bathtub and the time and rate of the water (dose) inputs that determine whether there will be any damage—not saturation of the drain capacity, i.e., elimination pathways, per se. Therefore, the argument put forth by Huff et al. (2010) is fallacious prima facie—it is a form of confusing necessity and sufficiency, also known as the fallacy of the converse.

## Conclusions

Kinetic data for ethylbenzene from rats and humans indicate a KMD range from 8 to 17 mg/L venous ethylbenzene and from 10 to 18 mg/L venous ethylbenzene, respectively, which corresponds to an inhalation concentration of approximately 200 ppm ethylbenzene. These KMDs, taken in a risk context, support the hypothesis that the neoplastic lesions seen in the NTP’s rodent cancer bioassay are a high-dose phenomenon secondary to saturation of elimination kinetics. The evidence indicates that typical human exposures to ethylbenzene, which are well below the KMD, are noncarcinogenic.

Our results also indicate that cancer endpoints measured in rodents exposed to ethylbenzene concentrations above our estimated KMD range are not relevant to toxicological testing or to risk assessments focused on protecting humans at typical human exposure levels. Thus, the evidence indicates that ethylbenzene is not a carcinogenic hazard for humans and does not pose a cancer risk to humans under foreseeable exposure conditions. Future work on this topic should either attempt to refute this hypothesis through better and higher quality studies or should focus on ethylbenzene effects below the KMD.

Risk assessors should consider the KMD when evaluating the mode of action that underlies dose–response relationships (Borgert et al. 2021; Burgoon et al. 2022) because, as we and others have discussed (Andersen 1981; Bus 2017), toxicity and modes of action are likely to change once exposure nears the point of metabolic saturation and/or saturation of clearance mechanisms. This is a critical point in the case of ethylbenzene risk assessment. At this time, the data clearly support the hypothesis that neoplastic lesions, and thus cancers, occur only when elimination kinetics are saturated. A kinetic threshold based on saturation of elimination should now be the default assumption for risk assessment of ethylbenzene.

Until and unless future work on ethylbenzene carcinogenesis convincingly falsifies that conclusion, research on the toxicity of ethylbenzene that is applicable for human risk assessment should be focused on ethylbenzene exposures that approximate foreseeable human exposure levels, which are more than three orders of magnitude below the KMD identified here. The most recent, comprehensive evaluation of ethylbenzene exposure (Kester and Morgott 2023) showed that in styrene production facilities, personal exposure concentrations to ethylbenzene collected over the 20-year period 2000 to 2020 were typically below the limit of detection (LoD). In contrast, the central tendency for styrene exposure levels was 0.1 ppm (434 µg/m^3^), with 1 ppm (4343 µg/m^3^) as the upper bound. These data indicate a high degree of conservatism in using styrene exposure levels in ethylbenzene/styrene production workers as an approximation of ethylbenzene exposure, and even greater conservatism when those data are used to estimate ethylbenzene exposure among the general population (Kester and Morgott 2023).

### Supplementary Information

Below is the link to the electronic supplementary material.

Supplementary file1 (DOCX 26 KB)

## Footnotes

1 For clarity, we avoid “pharmacokinetic” and “toxicokinetic” and instead use the terms “kinetics” or “kinetic processes” to reference the rates associated with chemical absorption, distribution, metabolism and elimination (ADME), irrespective of the dose range or qualitative nature of any biological effects.

## References

[CR1] Andersen ME (1981). Saturable metabolism and its relationship to toxicity. Crit Rev Toxicol.

[CR2] Ashby J, Brady A, Elcombe CR, Elliott BM, Ishmael J, Odum J, Tugwood JD, Kettle S, Purchase IFH (1994). Mechanistically based human hazard assessment of peroxisome proliferator-induced hepa-tocarcinogenesis. Hum Exp Toxicol.

[CR3] Borgert CJ, Fuentes C, Burgoon LD (2021). Principles of dose-setting in toxicology studies: the importance of kinetics for ensuring human safety. Arch Toxicol.

[CR4] Burgoon LD, Fuentes C, Borgert CJ (2022). A novel approach to calculating the kinetically derived maximum dose. Arch Toxicol.

[CR5] Bus JS (2017). “The dose makes the poison”: Key implications for mode of action (mechanistic) research in a 21st century toxicology paradigm. Curr Opin Toxicol.

[CR6] Charest-Tardif G, Tardif R, Krishnan K (2006). Inhalation pharmacokinetics of ethylbenzene in B6C3F1 mice. Toxicol Appl Pharmacol.

[CR7] Cohen SM, Zhongyu Y, Bus JS (2020). Relevance of mouse lung tumors to human risk assessment. J Toxicol Environ Health B Crit Rev.

[CR26] Cruzan G, Carlson GP, Johnson KA, Andrews LS, Banton MI, Bevan C (2002). Styrene respiratory tract toxicity and mouse lung tumors are mediated by CYP2F-generated metabolites. Regul Toxicol Pharmacol.

[CR8] Freundt KJ, Römer KG, Federsel RJ (1989). Decrease of inhaled toluene, ethyl benzene, m-xylene, or mesitylene in rat blood after combined exposure to ethyl acetate. Bull Environ Contam Toxicol.

[CR9] Golden R, Bus J, Calabrese E (2019). An examination of the linear no-threshold hypothesis of cancer risk assessment: Introduction to a series of reviews documenting the lack of biological plausibility of LNT. Chem Biol Interact.

[CR10] Haddad S, Tardif R, Charest-Tardif G, Krishnan K (1999). Physiological modeling of the toxicokinetic interactions in a quaternary mixture of aromatic hydrocarbons. Toxicol Appl Pharmacol.

[CR11] Haddad S, Charest-Tardif G, Tardif R, Krishnan K (2000). Validation of a physiological modeling framework for simulating the toxicokinetics of chemicals in mixtures. Toxicol Appl Pharmacol.

[CR12] Hard GC, Johnson KJ, Cohen SM (2009). A comparison of rat chronic progressive nephropathy with human renal disease-implications for human risk assessment. Crit Rev Toxicol.

[CR13] Huff J, Chan P, Melnick R (2010). Clarifying carcinogenicity of ethylbenzene. Regul Toxicol Pharmacol.

[CR14] IARC (2000). IARC Monographs on the Evaluation of the Carcinogenic Risk of Chemicals to Humans, vol 77, Some Industrial Chemicals.

[CR15] Kester JE, Morgott DA (2023). Ethylbenzene exposure in North America—an update. J Environ Exposure Assess.

[CR16] Marchand A, Aranda-Rodriguez R, Tardif R, Nong A, Haddad S (2015). Human inhalation exposures to toluene, ethylbenzene, and m-xylene and physiologically based pharmacokinetic modeling of exposure biomarkers in exhaled air, blood, and urine. Toxicol Sci.

[CR17] Nong A, Charest-Tardif G, Tardif R, Lewis DF, Sweeney LM, Gargas ML, Krishnan K (2007). Physiologically based modeling of the inhalation pharmacokinetics of ethylbenzene in B6C3F1 mice. J Toxicol Environ Health A.

[CR18] NTP (1999) (National Toxicology Program) NTP Technical Report on the Toxicology and Carcinogenesis Studies of Ethylbenzene (CAS NO. 100–41–4) in F344/N Rats and B6C3F1 Mice (Inhalation Studies) National Toxicology Program, Research Triangle Park, NC. 12579207

[CR19] Saghir SA (2010). In vitro metabolism and covalent binding of ethylbenzene to microsomal protein as a possible mechanism of ethylbenzene-induced mouse lung tumorigenesis. Regul Toxicol Pharmacol.

[CR20] Satopaa V, et al. (2011) Finding a ‘kneedle’ in a haystack: detecting knee points in system behavior. In: 2011 31st International Conference on Distributed Computing Systems Workshops. IEEE, Minneapolis, MN, USA, p 166–171

[CR21] Slikker W, Andersen ME, Bogdanffy MS, Bus JS, Cohen SD, Conolly RB, David RM, Doerrer NG, Dorman DC, Gaylor DW, Hattis D, Rogers JM, Setzer RW, Swenberg JA, Wallace K (2004). Dose-dependent transitions in mechanisms of toxicity. Toxicol Appl Pharmacol.

[CR22] Stott WT, Johnson KA, Bahnemann R, Day SJ, McGuirk RJ (2003). Evaluation of potential modes of action of inhaled ethylbenzene in rats and mice. Toxicol Sci.

[CR23] Tan YM, Barton HA, Boobis A, Brunner R, Clewell H, Cope R (2021). Opportunities and challenges related to saturation of toxicokinetic processes: implications for risk assessment. Regul Toxicol Pharmacol.

[CR24] Tardif R, Charest-Tardif G, Brodeur J, Krishnan K (1999). Physiologically based pharmacokinetic modeling of a ternary mixture of alkyl benzenes in rats and humans. Toxicol Appl Pharmacol.

[CR25] Wood J (2014). Trap of trends to statistical significance: likelihood of near significant P value becoming more significant with extra data. BMJ.

