# Supplementary material for: Kinetically-derived maximal dose (KMD) indicates lack of human carcinogenicity of ethylbenzene
Source: Arch Toxicol. 2023 Dec 7;98(1):327–34. doi: 10.1007/s00204-023-03629-7 (PMC10761441; doi:10.1007/s00204-023-03629-7)
Supplement: Supplementary file 1 — Supplementary file1 (DOCX 26 KB) [file 204_2023_3629_MOESM1_ESM.docx]

**Literature Evaluated for Data to Support a KMD Model for Ethylbenzene**

1. **Ethylbenzene [MAK Value Documentation, 2012**]. 2015 The MAK‐Collection for Occupational Health and Safety. ed. American Cancer Society. 1–18.
2. **Agency for Toxic Substances and Disease Registry**. 2010. Toxicological profile for ethylbenzene.
3. **Astrand I, Engström J, Ovrum P**. 1978. Exposure to xylene and ethylbenzene. I. Uptake, distribution and elimination in man. *Scand J Work Environ Health* **4**: 185–94.
4. **Berthet A, de Batz A, Tardif R, Charest-Tardif G, Truchon G, Vernez D, Droz PO**. 2010. Impact of biological and environmental variabilities on biological monitoring--an approach using toxicokinetic models. *J Occup Environ Hyg* **7**: 177–84.
5. **Campbell JLJ, Fisher JW**. 2007. A PBPK modeling assessment of the competitive metabolic interactions of JP-8 vapor with two constituents, m-xylene and ethylbenzene. *Inhal Toxicol* **19**: 265–73.
6. **Chan PC, Hasemani JK, Mahleri J, Aranyi C**. 1998. Tumor induction in F344/N rats and B6C3F1 mice following inhalation exposure to ethylbenzene. *Toxicol Lett* **99**: 23–32.
7. **Charest-Tardif G, Tardif R, Krishnan K**. 2006. Inhalation pharmacokinetics of ethylbenzene in B6C3F1 mice. *Toxicol Appl Pharmacol* **210**: 63–9.
8. **Cheng S, Bois FY**. 2011. A mechanistic modeling framework for predicting metabolic interactions in complex mixtures. *Environ Health Perspect* **119**: 1712–8.
9. **Chin BH, McKelvey JA, Tyler TR, Calisti LJ, Kozbelt SJ, Sullivan LJ**. 1980. Absorption, distribution, and excretion of ethylbenzene, ethylcyclohexane, and methylethylbenzene isomers in rats. *Bulletin of Environmental Contamination and Toxicology* **24**: 477–83.
10. **Cragg ST, Clarke EA, Daly IW, Miller RR, Terrill JB, Ouellette RE**. 1989. Subchronic inhalation toxicity of ethylbenzene in mice, rats, and rabbits. *Fundam Appl Toxicol* **13**: 399–408.
11. **Davidson CJ, Hannigan JH, Bowen SE**. 2021. Effects of inhaled combined Benzene, Toluene, Ethylbenzene, and Xylenes (BTEX): Toward an environmental exposure model. *Environ Toxicol Pharmacol* **81**: 103518.
12. **Dennison JE, Andersen ME, Clewell HJ, Yang RS**. 2004. Development of a physiologically based pharmacokinetic model for volatile fractions of gasoline using chemical lumping analysis. *Environ Sci Technol* **38**: 5674–81.
13. **Dennison JE, Andersen ME, Dobrev ID, Mumtaz MM, Yang RS**. 2004. PBPK modeling of complex hydrocarbon mixtures: gasoline. *Environ Toxicol Pharmacol* **16**: 107–19.
14. **Dennison JE, Andersen ME, Yang RS**. 2003. Characterization of the pharmacokinetics of gasoline using PBPK modeling with a complex mixtures chemical lumping approach. *Inhal Toxicol* **15**: 961–86.
15. **Dennison JE, Bigelow PL, Mumtaz MM, Andersen ME, Dobrev ID, Yang RS**. 2005. Evaluation of potential toxicity from co-exposure to three CNS depressants (toluene, ethylbenzene, and xylene) under resting and working conditions using PBPK modeling. *J Occup Environ Hyg* **2**: 127–35.
16. **Dutkiewicz T, Tyras H**. 1967. A study of the skin absorption of ethylbenzene in man. *Br J Ind Med* **24**: 330–2.
17. **EL MASRY AM, SMITH JN, WILLIAMS RT**. 1956. Studies in detoxication. 69. The metabolism of alkylbenzenes: n-propylbenzene and n-butylbenzene with further observations on ethylbenzene. *Biochem J* **64**: 50–6.
18. **Elovaara E, Engström K, Nickels J, Aito A, Vainio H**. 1985. Biochemical and morphological effects of long-term inhalation exposure of rats to ethylbenzene. *Xenobiotica* **15**: 299–308.
19. **Elovaara E, Engström K, Vainio H**. 1984. Metabolism and disposition of simultaneously inhaled m-xylene and ethylbenzene in the rat. *Toxicol Appl Pharmacol* **75**: 466–78.
20. **Engström J, Bjurström R**. 1978. Exposure to xylene and ethylbenzene. II. Concentration in subcutaneous adipose tissue. *Scand J Work Environ Health* **4**: 195–203.
21. **Engström K, Elovaara E, Aitio A**. 1985. Metabolism of ethylbenzene in the rat during long-term intermittent inhalation exposure. *Xenobiotica* **15**: 281–6.
22. **Engström K, Riihimäki V, Laine A**. 1984. Urinary disposition of ethylbenzene and m-xylene in man following separate and combined exposure. *Int Arch Occup Environ Health* **54**: 355–63.
23. **Faber WD, Roberts LSG, Stump DG, Beck M, Kirkpatrick D, Regan KS, Tort M, Moran E, Banton M**. 2007. Inhalation developmental neurotoxicity study of ethylbenzene in Crl-CD rats. *Birth Defects Res B Dev Reprod Toxicol* **80**: 34–48.
24. **Faber WD, Roberts LSG, Stump DG, Tardif R, Krishnan K, Tort M, Dimond S, Dutton D, Moran E, Lawrence W**. 2006. Two generation reproduction study of ethylbenzene by inhalation in Crl-CD rats. *Birth Defects Res B Dev Reprod Toxicol* **77**: 10–21.
25. **Fabian E, Bordag N, Herold M, Kamp H, Krennrich G, Looser R, Ma-Hock L, Mellert W, Montoya G, Peter E, Prokudin A, Spitzer M, Strauss V, Walk T, Zbranek R, van Ravenzwaay B**. 2016. Metabolite profiles of rats in repeated dose toxicological studies after oral and inhalative exposure. *Toxicol Lett* **255**: 11–23.
26. **Fechter LD, Gearhart C, Fulton S, Campbell J, Fisher J, Na K, Cocker D, Nelson-Miller A, Moon P, Pouyatos B**. 2007. Promotion of noise-induced cochlear injury by toluene and ethylbenzene in the rat. *Toxicol Sci* **98**: 542–51.
27. **Freundt KJ, Römer KG, Federsel RJ**. 1989. Decrease of inhaled toluene, ethyl benzene, m-xylene, or mesitylene in rat blood after combined exposure to ethyl acetate. *Bull Environ Contam Toxicol* **42**: 495–8.
28. **Fuciarelli AF**. 2000. Ethylbenzene two-week repeated-dose inhalation toxicokinetic study report.
29. **Gamberale F, Annwall G, Hultengren M**. 1978. Exposure to xylene and ethylbenzene. III. Effects on central nervous functions. *Scand J Work Environ Health* **4**: 204–11.
30. **Haddad S, Charest-Tardif G, Tardif R, Krishnan K**. 2000. Validation of a physiological modeling framework for simulating the toxicokinetics of chemicals in mixtures. *Toxicol Appl Pharmacol* **167**: 199–209.
31. **Haddad S, Tardif R, Charest-Tardif G, Krishnan K**. 1999. Physiological modeling of the toxicokinetic interactions in a quaternary mixture of aromatic hydrocarbons. *Toxicol Appl Pharmacol* **161**: 249–57.
32. **IARC Working Group on the Carcinogenic Risks to Humans**. 2000. Ethylbenzene. ed. Some industrial chemicals/IARC monographs on the evaluation of carcinogenic risk to humans #77. Lyon, France: IARC.
33. **Ishii S, Katagiri R, Kitamura K, Shimojima M, Wada T**. 2017. Evaluation of the ECETOC TRA model for workplace inhalation exposure to ethylbenzene in Japan. *Journal of Chemical Health & Safety J Chem Health Saf* **24**: 8–20.
34. **Marchand A, Aranda-Rodriguez R, Tardif R, Nong A, Haddad S**. 2015. Human inhalation exposures to toluene, ethylbenzene, and m-xylene and physiologically based pharmacokinetic modeling of exposure biomarkers in exhaled air, blood, and urine. *Toxicol Sci* **144**: 414–24.
35. **Marchand A, Aranda-Rodriguez R, Tardif R, Nong A, Haddad S**. 2016. Evaluation and modeling of the impact of coexposures to VOC mixtures on urinary biomarkers. *Inhal Toxicol* **28**: 260–73.
36. **Mellert W, Deckardt K, Kaufmann W, van Ravenzwaay B**. 2007. Ethylbenzene: 4-and 13-week rat oral toxicity. *Archives of toxicology* **81**: 361–70.
37. **National Toxicology Program**. 1992. Ethylbenzene (CAS No. 100-41-4) in F344/N rats and B6C3F1 mice (inhalation studies). *NTP Technical Report* **10**:
38. **National Toxicology Program**. 1999. NTP technical report on the toxicology and carcinogenesis studies of ethylbenzene (CAS No. 100-41-4) in F344/N rats and B6C3F1 mice. *NTP Technical Report* **466**:
39. **Nielsen, E,, Ladefoged, O**. 2013. Evaluation of health hazards by exposure to ethylbenzene and proposal of a health-based quality criterion for ambient air. The Danish Environmental Protection Agency.
40. **Nong A, Charest-Tardif G, Tardif R, Lewis DF, Sweeney LM, Gargas ML, Krishnan K**. 2007. Physiologically based modeling of the inhalation pharmacokinetics of ethylbenzene in B6C3F1 mice. *J Toxicol Environ Health A* **70**: 1838–48.
41. **Römer KG, Federsel RJ, Freundt KJ**. 1986. Rise of inhaled toluene, ethyl benzene, m-xylene, or mesitylene in rat blood after treatment with ethanol. *Bull Environ Contam Toxicol* **37**: 874–6.
42. **Ruiz P, Emond C, McLanahan ED, Joshi-Barr S, Mumtaz M**. 2020. Exploring Mechanistic Toxicity of Mixtures Using PBPK Modeling and Computational Systems Biology. *Toxicol Sci* **174**: 38–50.
43. **Saghir SA, Rick DL, McClymont EL, Zhang F, Bartels MJ, Bus JS**. 2009. Mechanism of ethylbenzene-induced mouse-specific lung tumor: metabolism of ethylbenzene by rat, mouse, and human liver and lung microsomes. *Toxicol Sci* **107**: 352–66.
44. **Saillenfait AM, Gallissot F, Morel G, Bonnet P**. 2003. Developmental toxicities of ethylbenzene, ortho-, meta-, para-xylene and technical xylene in rats following inhalation exposure. *Food Chem Toxicol* **41**: 415–29.
45. **Saillenfait AM, Gallissot F, Sabaté JP, Bourges-Abella N, Cadot R, Morel G, Lambert AM**. 2006. Developmental toxicity of combined ethylbenzene and methylethylketone administered by inhalation to rats. *Food Chem Toxicol* **44**: 1287–98.
46. **Saillenfait AM, Gallissot F, Sabate JP, Bourges-Abella N, Muller S**. 2007. Developmental toxic effects of ethylbenzene or toluene alone and in combination with butyl acetate in rats after inhalation exposure. *J Appl Toxicol* **27**: 32–42.
47. **Sams C, Loizou GD, Cocker J, Lennard MS**. 2004. Metabolism of ethylbenzene by human liver microsomes and recombinant human cytochrome P450s (CYP). *Toxicol Lett* **147**: 253–60.
48. **Sterner TR, Robinson PJ, Hack CE, Qi L, Narayanan L, Law ST, Covington TR, Merrill EA, Grobe N, Brown DN, Mattie DR**. 2017. Jet fuel exacerbated noise-induced hearing loss: focus on prediction of central auditory processing dysfunction.
49. **Stott WT, Johnson KA, Bahnemann R, Day SJ, McGuirk RJ**. 2003. Evaluation of potential modes of action of inhaled ethylbenzene in rats and mice. *Toxicol Sci* **71**: 53–66.
50. **Sweeney LM, Kester JE, Kirman CR, Gentry PR, Banton MI, Bus JS, Gargas ML**. 2015. Risk assessments for chronic exposure of children and prospective parents to ethylbenzene (CAS No. 100-41-4). *Crit Rev Toxicol* **45**: 662–726.
51. **Take M, Takeda T, Ishikawa H, Matsumoto M, Nagano K, Fukushima S**. 2020. Area under the blood concentration-time curve (AUC) of ethylbenzene concentration in rats: relationship to inhalation and oral administration route-dose. *J Environ Sci Health A Tox Hazard Subst Environ Eng* **55**: 1596–603.
52. **Tardif R, Charest-Tardif G, Brodeur J, Krishnan K**. 1997. Physiologically based pharmacokinetic modeling of a ternary mixture of alkyl benzenes in rats and humans. *Toxicol Appl Pharmacol* **144**: 120–34.
53. **Tohon H, Valcke M, Haddad S**. 2019. An assessment of the impact of multi-route co-exposures on human variability in toxicokinetics: A case study with binary and quaternary mixtures of volatile drinking water contaminants. *J Appl Toxicol* **39**: 974–91.
54. **Ungváry G, Tátrai E**. 1985. On the embryotoxic effects of benzene and its alkyl derivatives in mice, rats and rabbits. *Arch Toxicol Suppl* **8**: 425–30.
55. **Valcke M, Haddad S**. 2015. Assessing human variability in kinetics for exposures to multiple environmental chemicals: a physiologically based pharmacokinetic modeling case study with dichloromethane, benzene, toluene, ethylbenzene, and m-xylene. *J Toxicol Environ Health A* **78**: 409–31.
56. **Yuan W, Sequeira DJ, Cawley GF, Eyer CS, Backes WL**. 1997. Time course for the modulation of hepatic cytochrome P450 after administration of ethylbenzene and its correlation with toluene metabolism. *Arch Biochem Biophys* **339**: 55–63.
57. **Zhang M, Wang Y, Wang X, Liu J, Zhang J, Gu Q**. 2016. Roles of oxidative stress, apoptosis, and heme oxygenase-1 in ethylbenzene-induced renal toxicity in NRK-52E cells. *Toxicol Ind Health* **32**: 1952–60.
58. **Zhang M, Wang YR, Wang Q, Yang JY, Yang DY, Liu J, Li JG**. 2010. Involvement of Mitochondria-Mediated Apoptosis in Ethylbenzene-Induced Renal Toxicity in Rat. *TOXICOLOGICAL SCIENCES* **115**: 295–303.
